# A data visualisation method for assessing exposure misclassification in case-crossover studies: the example of tricyclic antidepressants and the risk of hip fracture in older people

**DOI:** 10.1186/s12874-021-01230-z

**Published:** 2021-02-27

**Authors:** Michael J. Leach, Elizabeth E. Roughead, Nicole L. Pratt

**Affiliations:** 1 Quality Use of Medicines and Pharmacy Research Centre (QUMPRC), UniSA Clinical and Health Sciences, CEA-19, University of South Australia, Adelaide, SA 5001 Australia; 2 School of Rural Health, Monash University, 26 Mercy Street, Bendigo, VIC 3550 Australia

**Keywords:** Case-crossover study, Data visualisation, Elderly, Exposure misclassification, Hip fracture, Pharmacoepidemiology, Tricyclic antidepressants

## Abstract

**Background:**

The case-crossover design is suited to medication safety studies but is vulnerable to exposure misclassification. Using the example of tricyclic antidepressants and the risk of hip fracture, we present a data visualisation tool for observing exposure misclassification in case-crossover studies.

**Methods:**

A case-crossover study was conducted using Australian Government Department of Veterans’ Affairs claims data. Beneficiaries aged over 65 years who were hospitalised for hip fracture between 2009 and 2012 were included. The case window was defined as 1–50 days pre fracture. Control window one and control window two were defined as 101–150 and 151–200 days pre fracture, respectively. Patients were stratified by whether exposure status changed when control window two was specified instead of control window one. To visualise potential misclassification, each subject’s tricyclic antidepressant dispensings were plotted over the 200 days pre fracture.

**Results:**

The study population comprised 8828 patients with a median age of 88 years. Of these subjects, 348 contributed data to the analyses with either control window. The data visualisation suggested that 14% of subjects were potentially misclassified with control window one while 45% were misclassified with control window two. The odds ratio for the association between tricyclic antidepressants and hip fracture was 1.18 (95% confidence interval = 0.91–1.52) using control window one, whereas risk was significantly increased (odds ratio = 1.43, 95% confidence interval = 1.11–1.83) using control window two.

**Conclusions:**

Exposure misclassification was less likely to be present with control window one than with an earlier control window, control window two. When specifying different control windows in a case-crossover study, data visualisation can help to assess the extent to which exposure misclassification may contribute to variable results.

## Background

The case-crossover design is a within-person method that quantifies the effects of intermittent exposures on acute outcomes, while minimising confounding bias and overcoming control selection issues [1]. The case-crossover design compares each individual’s exposure status immediately before an outcome (the case window) with their own exposure status at an earlier stage or stages in recent history (the control window). This means that each case serves as his or her own control. As such, effect estimates obtained using a case-crossover design inherently control for patient-specific factors that do not vary within individuals between the chosen control and case windows [1]. The validity of case-crossover study results depends on the following five assumptions: at least some intermittent medicine use, absence of carryover effects between control and case windows, a stable baseline risk of the outcome, stable prevalence of medicine use in the underlying population, and an acute-onset outcome [1–4].

Although the case-crossover design overcomes bias relating to selecting control patients, there may still be bias when choosing a control window among case patients. The risk estimates obtained from a case-crossover analysis depend on the choice of case and control windows. This is reflected in the underlying assumption of biologically plausible exposure windows with no carryover effects from the control window to the case window [5]. Published guidelines for pharmacoepidemiological case-crossover studies recommend conducting sensitivity analyses with alternative case and control windows as well as reporting the numbers of discordant cases across primary and sensitivity analyses [5]. A discordant case is a subject exposed during the case window only (and not in the control window) or a subject exposed in the control window only (and not in the case window). The ratio of the number of persons exposed in the case window only to the number of persons exposed in the control window only gives the odds ratio (OR) for the case-crossover analysis. This OR estimates the relative risk of the outcome in exposed versus unexposed time [6]. As the OR depends on the numbers of discordant cases and the choice of exposure windows, there is a need to develop methods for assessing exposure classification.

The assessment of tricyclic antidepressant (TCA) use and the risk of hip fracture is an example of an analysis that meets the underlying assumptions of the case-crossover design, including the assumption of at least some intermittent exposure in instances when TCAs are used by older people to treat short-term neuropathic pain [7, 8]. In a previously-published case-crossover study of the risk of hip fracture associated with TCAs in older people, we defined the lengths of the case window, washout period and control window as the period of time within which 75% of patients in our dataset had a repeat TCA dispensing (i.e. 50 days). The washout period was included to minimise carry-over effects; however, it is possible that a script dispensed during one window would carry over to the next. We found no association between TCAs and hip fracture when the case window was set as 1–50 days pre fracture and the control window as 101–150 days pre fracture [7]. When the control window definition was changed to an earlier period of 151–200 days pre fracture, however, an association was found [7]. Such discrepant results may indicate the potential for misclassification bias.

A potential means of elucidating exposure misclassification in pharmacoepidemiological case-crossover studies is data visualisation. A data visualisation is any visual tool that enables one to view and better understand a dataset’s underlying structure [9]. In pharmacoepidemiology, a range of data visualisation tools (e.g. paling palettes illustrating numbers of patients as stick figures) have been used to show data relating to the risk-benefit assessment of medicines [10]. With regard to pharmacoepidemiological case-crossover studies, one known study has used data visualisation to visually assess the impact of varying exposure window definitions. In a Canadian case-crossover study of drugs for attention deficit hyperactivity disorder and the risk of cardiovascular disease, stars-and-stripes diagrams of dispensing dates (stars) and days supply (stripes) were created using administrative claims data [11]. These stars-and-stripes diagrams visualised the trade-offs between the specificity and sensitivity of different exposure window definitions as well as the impact of varying exposure window widths on OR estimates. As this study was published as an abstract without the data visualisation, however, there is still no established method for assessing the potential for exposure misclassification across alternative exposure windows in case-crossover designs.

The present study aimed to develop and demonstrate a novel data visualisation method for conducting an a posteriori assessment of the extent of exposure misclassification in the case-crossover design, using the previously-published example of risk of hip fracture associated with TCAs [7].

## Methods

The case-crossover method used in this study has been described previously [7]. Briefly, data were sourced from the Australian Government Department of Veterans’ Affairs healthcare claims database to investigate psychoactive medicine use and the risk of hip fracture among older people. The subjects were Department of Veterans’ Affairs beneficiaries aged over 65 years who were hospitalised for a hip fracture (International Classification of Diseases and Related Health Problems-10-Australian Modification [12] codes S72.0 and S72.1) between 2009 and 2012. The case window was defined as 1–50 days pre fracture. Two alternative control windows were specified. Control window one (CW1) was defined as 101–150 days pre fracture, with a corresponding washout period of 51–100 days pre fracture, while control window two (CW2) was defined as 151–200 days pre fracture, with a corresponding washout period of 51–150 days pre fracture. A subject was classified based on the dispensing of TCAs (Anatomical Therapeutic Chemical [13] classification code N06AA) during the case and control windows. A univariable conditional logistic regression model was used to estimate an OR and 95% confidence interval (CI) for the association between TCAs and hip fracture, with individual subjects acting as strata.

Following this case-crossover study, a data visualisation approach was developed to compare the potential for misclassification of TCA exposure when the choice of control window was varied. For each one of 348 individuals (y-axis), TCA dispensings were plotted on a time line over the 200-day time period before hip fracture (x-axis). To reveal misclassification that could affect the calculation of the OR, patients were stratified based on whether their exposure status changed or remained the same when CW2 was specified rather than CW1. The different exposure statuses were intermittent users (exposed in the case window but not the control window), recent stoppers (exposed in the control window but not the case window), continuous users (exposed in both the case and control windows), and non-users (exposed in neither the case window nor the control window). To determine the extent of misclassification bias among these four groups, six strata featuring a change in exposure status were created for the data visualisation: recent stoppers becoming non-users (Stratum A), non-users becoming recent stoppers (Stratum B), recent stoppers remaining recent stoppers (Stratum C), intermittent users becoming continuous users (Stratum D), continuous users becoming intermittent users (Stratum E), and intermittent users remaining intermittent users (Stratum F). Two additional strata were not assessed due to the lack of a change in exposure status. Within each stratum assessed, the potential for exposure misclassification was determined by sorting and visually inspecting dispensing histories. The dispensing histories of persons in Stratum A, Stratum B, and Stratum C were sorted to show the latest dispensing closest to the case window, as these patients were unexposed in the case window. The dispensing histories of persons in Stratum D, Stratum E, and Stratum F were sorted to show the earliest dispensing closest to the control window(s), as these patients were unexposed in the control window(s). The timing of a dispensing close to a window in which the patient was categorised as unexposed suggests potential for exposure misclassification.

Correct classification and misclassification were defined in terms of clinical consensus decision making informed by data visualisation. For each stratum, dispensing histories were visually inspected to determine the percentages of patients who had been correctly classified and potentially misclassified. The visualisation of the data enabled consensus decisions regarding correct classification and misclassification among three researchers with backgrounds in pharmacy, statistics, and pharmacoepidemiology. Any disagreements were resolved through inclusive discussion with reference to the data visualisation. Misclassification was determined by considering the quantity of dispensings in the washout period as well as the clustering of dispensings close together on either side of an exposure window or washout period. Table 1 shows the rules used to determine misclassification and correct classification of TCA exposure across all strata. These rules were based on the idea that a dispensing close to an exposure window in which the case was said to be unexposed suggests the potential for exposure misclassification. For each of the different control windows, the percentage of people correctly classified was calculated by summing the percentages of the sample in strata considered to be correctly classified.

**Table 1:** Rules used to determine correct classification and misclassification of TCA exposure status across all strata

**Stratum**	**Stratum Definition**	**Rule**
A	Recent stoppers with CW1 becoming non-users with CW2	If greater than half of the latest prescriptions were closer to the RHS of CW1 than the LHS of CW1, then correct classification with CW1 and misclassification with CW2.
B	Non-users with CW1 becoming recent stoppers with CW2	If greater than half of the people had a dispensing in the washout period, then misclassification with CW1 and correct classification with CW2. Those exposed in CW2 only were considered to be misclassified as recent stoppers due to the 150-day gap before hip fracture.
C	Recent stoppers with CW1 remaining recent stoppers with CW2	If greater than half of the latest prescriptions were closer to the RHS of CW1 than the LHS of CW2, then correct classification in both CW1 and CW2.
D	Intermittent users with CW1 becoming continuous users with CW2	If greater than half of the latest prescriptions were closer to the RHS of CW2 than the LHS of CW2, then misclassification with CW1 and correct classification with CW2.
E	Continuous users with CW1 becoming intermittent users with CW2	If greater than half of prescriptions were closer to the LHS of CW1 than the RHS of CW1, then correct classification with CW1 and misclassification with CW2.
F	Intermittent users with CW1 remaining intermittent users with CW2	If greater than half of the earliest prescriptions were located in the case window rather than the washout period, then correct classification in both CW1 and CW2.

*CW1* Control window 1, *CW2* Control window 2, *RHS* Right-hand side, *LHS* Left-hand side

The case-crossover analyses were performed, and the data visualisation was created, using SAS version 9.4 (SAS Institute, Inc., Cary, NC).

## Results

The total cohort comprised 8828 patients with a median age of 88 years. Sixty-three percent of patients were women. All characteristics are presented in Table 2. Table 3 shows the numbers of patients contributing to the case-crossover analysis using either control window definition, alongside the OR and 95% CI.

**Table 2:** Characteristics of older patients who were admitted to hospital for a hip fracture between 2009 and 2012

**Characteristic**	**No. of patients (*n* = 8828)**
Female gender	5592 (63%)
Median age (Q1-Q3) [years]	88 (85–91)
Residential Location	
Major cities	4999 (57%)
Inner regional	3068 (35%)
Outer regional	708 (7.9%)
Remote	53 (0.6%)
Unknown	7 (0.08%)
Socioeconomic Status	
Upper	2537 (28%)
Middle-upper	1559 (18%)
Middle	1664 (19%)
Lower-middle	1737 (20%)
Lower	1320 (15%)
Unknown	11 (0.1%)
Residential Status	
Community domicile	5668 (64%)
Residential aged care	3160 (36%)

*n* Number, *Q1* Lower quartile (25th percentile), *Q3* Upper quartile (75th percentile)

**Table 3:** TCA exposure classification and effect estimates with alternative control windows (*n* = 8828)

**Control Window**	**TCA Exposure Classification**				**OR**^**c**^ **(95% CI)**
	**Continuous Users**^**a**^	**Non-Users**^**a**^	**Intermittent Users**^**b**^	**Recent Stoppers**^**b**^	
1^d^	349	8244	127	108	1.18 (0.91–1.52)
2^e^	326	8247	150	105	1.43 (1.11–1.83)

Adapted from [7]

*TCA* Tricyclic antidepressant, *OR* Odds ratio, *CI* Confidence interval

^a^Excluded from analysis due to concordant exposure between the case window and the control window

^b^Included in analysis due to discordant exposure between the case window and the control window

^c^OR for intermittent exposure compared with recent stopping

^d^A control window of 101–150 days pre-fracture

^e^A control window of 151–200 days pre-fracture

**Fig. 1:**
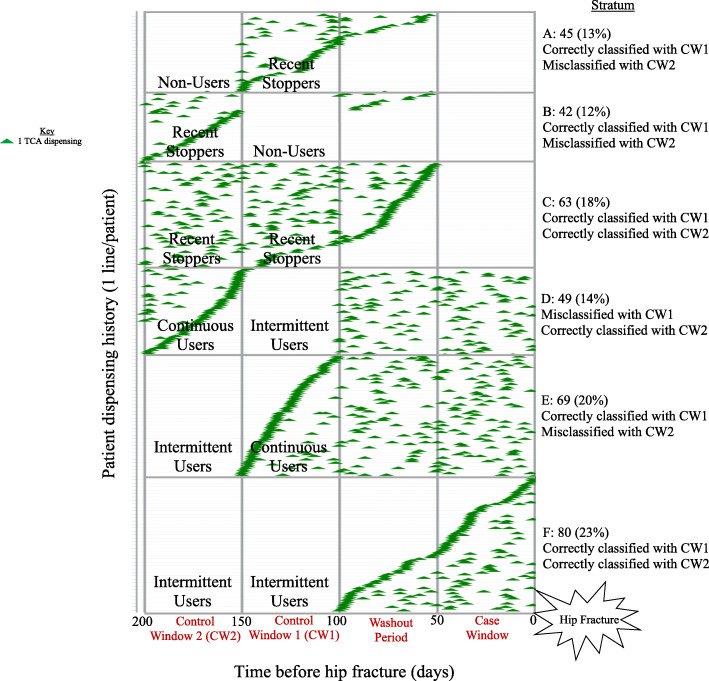
Data visualisation showing correct classification and misclassification of TCA exposure (*n* = 348). TCA – tricyclic antidepressant. A – Changed from recent stoppers with control window 1 to non-users with control window 2. B – Changed from non-users with control window 1 to recent stoppers with control window 2. C – Remained recent stoppers with control window 2. D – Changed from intermittent users with control window 1 to continuous users with control window 2. E – Changed from continuous users with control window 1 to intermittent users with control window 2. F – Remained intermittent users with control window 2

Overall, 348 unique subjects contributed data to the case-crossover analysis using either control window definition. There was an overlap in the patients included with each definition. Figure 1 shows TCA dispensing during the 200 days before hip fracture for patients who were classified as intermittent users or recent stoppers in the case-crossover designs with CW1 and CW2. Disagreements that arose among the three researchers on the classifications of continuous users (Fig. 1; Strata D and E) were resolved through inclusive discussions and consensus decision making. Overall, using CW1, 86% of patients appeared to be correctly classified (Fig. 1; Strata A, B, C, E and F) in the data visualisation while 14% of patients appeared to be misclassified (Fig. 1; Stratum D). Using CW2, 55% of patients appeared to be correctly classified (Fig. 1; Strata C, D and F) while 45% appeared to be misclassified (Fig. 1; Strata A, B and E).

## Discussion

In this case-crossover study of the association between TCAs and hip fracture, we identified that the choice of control window has the potential to introduce exposure misclassification. In particular, specifying a control window further away from the case window misclassified continuous TCA users as intermittent TCA users (Stratum E), giving a persistent user bias evident through data visualisation [14]. For most of the patients in Stratum E, continuous use is more likely than intermittent use because the earliest time of TCA dispensing is clustered closer to the beginning of CW1 than to the end of CW1. The analysis with CW1, which was closer to the case window, likely provides a less biased result and suggests that there is no association between TCAs and hip fracture. The significant result obtained with the earlier control window, CW2, may be partly explained by unmeasured patient-specific, time-varying confounding factors such as alcohol intake and concomitant use of other psychoactive drugs. While a meta-analysis of case-control and cohort studies suggests an association between TCAs and hip fracture, this relationship has not been previously assessed in any other case-crossover study controlling for patient-specific, time-invariant confounders [15]. In line with Maclure and Mittleman’s recommendation [6] to conduct a case-control study as a type of validation for a case-crossover study, our case-control study conducted in the same setting found that new use of TCAs was not independently associated with hip fracture [16]. The conservative use of TCAs at low doses to treat neuropathic pain or depression in older people could partly explain the lack of association in our case-crossover and case-control studies [7].

While the case-crossover design was originally developed to circumvent the issue of control selection when studying myocardial infarction triggers [6], it has since been widely used to investigate triggers (as opposed to chronic risk factors) in various other areas, including pharmacoepidemiology [5, 6]. Our proposed data visualisation helps to determine the potential for misclassification of exposure in a pharmacoepidemiological case-crossover study. This data visualisation approach may help validate the case-crossover assumption of biologically plausible exposure windows without carryover effects [5] and offers an alternative to the stars-and-stripes diagram, which was used to visualise trade-offs between sensitivity and specificity in a case-crossover study of attention deficit hyperactivity disorder drugs and the risk of cardiovascular disease [11]. Regardless of the technique used, it is advisable to visualise dispensing histories across alternative exposure windows when employing a case-crossover study, with a view to determining the validity of the exposure window definitions. The potential for misclassification bias should always be assessed alongside the five assumptions underlying the case-crossover study [1–4].

A limitation of the data visualisation method proposed here is the subjectivity involved in distinguishing between correct classification and misclassification. This issue may be partially addressed through our approach: applying a rule based on the usual medicine exposure duration (e.g. setting window lengths as the time for 75% of patients to have a repeat dispensing), before aiming for consensus decisions regarding correct classification and misclassification within the visualisation. Another limitation is that a small number of patients in Stratum B (Fig. 1) had a dispensing in the washout period and, thus, may not have in fact been non-users. Based on the overall observed pattern of dispensing, however, misclassification is minimised for the Stratum B group as a whole when they are classified as non-users with CW1 rather than recent stoppers with CW2. While there was evidence of short-term use of TCAs in patients’ dispensing histories, we lacked data on indications such as neuropathic pain and, therefore, were unable to fully assess the case-crossover assumption of intermittent exposure. Additionally, given that dispensing was considered a marker of medicine use, non-adherence was a source of potential misclassification not considered here. The issue of differential recall bias described previously [17] was not problematic, however, due to the use of administrative claims data. Lastly, there is the potential for this visualisation to be too big in certain case-crossover applications. While the method provides a useful visualisation with discriminable data points for 348 subjects, it may not be useful in a big data context with tens of thousands of cases.

Future work could focus on developing interactive data visualisations for assessing exposure misclassification under various scenarios and assumptions, with a view to more rigorously and clearly exploring the impact of misclassification on the results of pharmacoepidemiological case-crossover studies.

## Conclusions

The choice of a control window is a critical component of the design of a case-crossover study. In our prior case-crossover study [7], variation in the choice of control window led to differing results for the association between TCAs and hip fracture. Our proposed data visualisation approach revealed that TCA exposure misclassification was less likely to be present in the case-crossover analysis with a control window closer in time to the case window than the analysis with an earlier control window. This suggest that the results of the analysis with the control window closer in time to the case window, which suggested no association between TCAs and hip fracture, is more likely to be valid. When assessing different control windows in a case-crossover study leads to variable results, data visualisation is likely to be useful for assessing the extent to which exposure misclassification contributes to those results.

## Data Availability

The data that support the findings of this study were provided by the Australian Government Department of Veterans’ Affairs. The data that support the findings of this study are available from the Australian Government Department of Veterans’ Affairs but restrictions apply to the availability of these data, which were used under license for the current study, and so are not publicly available. Data may, however, be provided upon reasonable request to and permission from the Australian Government Department of Veterans’ Affairs.

## Abbreviations

CI: Confidence interval
CW1: Control window one
CW2: Control window two
n: Number
OR: Odds ratio
Q1: Lower quartile (25th percentile)
Q3: Upper quartile (75th percentile)
TCA: Tricyclic antidepressant

## References

[1] Maclure M (1991). The case-crossover design: a method for studying transient effects on the risk of acute events. Am J Epidemiol.

[2] Delaney J, Suissa S (2009). The case-crossover study design in pharmacoepidemiology. Stat Methods Med Res.

[3] Maclure M, Fireman B, Nelson JC, Hua W, Shoaibi A, Paredes A (2012). When should case-only designs be used for safety monitoring of medical products?. Pharmacoepidemiol Drug Saf.

[4] Mittleman M, Mostofsky E (2014). Exchangeability in the case-crossover design. Int J Epidemiol.

[5] Consiglio G, Burden AM, Maclure M, McCarthy L, Cadarette S (2013). Case-crossover study design in pharmacoepidemiology: systematic review and recommendations. Pharmacoepidemiol Drug Saf.

[6] Maclure M, Mittleman MA (2000). Should we use a case-crossover design?. Annu Rev Publ Health.

[7] Leach MJ, Pratt NL, Roughead EE (2015). Psychoactive medicine use and the risk of hip fracture in older people: a case-crossover study. Pharmacoepidemiol Drug Saf.

[8] Rossi S (2017). Australian medicines handbook 2017.

[9] Unwin A, Chen C, Hardle W, Chen C, Hardle W, Unwin A (2008). Introduction. Handbook of data visualization.

[10] Mt-Isa S, Hallgreen CE, Asiimwe A, Downey G, Genov G, Hermann R (2013). Review of visualisation methods for the representation of benefit-risk assessment of medication: stage 2 of 2.

[11] Maclure M, Morrow R, Carney G, Dormuth C (2011). 'Stars and stripes’ diagrams of discordant cases’ exposures in case-crossover studies using healthcare databases. Pharmacoepidemiol Drug Saf.

[12] National Centre for Classification in Health. The international statistical classification of diseases and related health problems, 10th revision, Australian modification (ICD-10-AM). Sydney: University of Sydney; 2005. 10169442

[13] World Health Organization Collaborating Centre for Drug Statistics Methodology, ATC/DDD Index 2014; 2014. https://www.whocc.no/atc_ddd_index/. Accessed 7 Mar 2014.

[14] Pottegard A, Hallas J (2013). Assigning exposure duration to single prescriptions by use of the waiting time distribution. Pharmacoepidemiol Drug Saf.

[15] Oderda L, Young JR, Asche CV, Pepper GA (2012). Psychotropic-related hip fractures: meta-analysis of first-generation and second-generation antidepressant and antipsychotic drugs. Ann Pharmacother.

[16] Leach MJ, Pratt NL, Roughead EE (2017). The risk of hip fracture due to mirtazapine exposure when switching antidepressants or using other antidepressants as add-on therapy. Drugs Real World Outcomes.

[17] Marshall RJ, Jackson RT (1993). Analysis of case-crossover designs. Stat Med.

